# Self‐Organized Fullerene Interfacial Layer for Efficient and Low‐Temperature Processed Planar Perovskite Solar Cells with High UV‐Light Stability

**DOI:** 10.1002/advs.201700018

**Published:** 2017-04-19

**Authors:** Jiangsheng Xie, Xuegong Yu, Jiabin Huang, Xuan Sun, Yunhai Zhang, Zhengrui Yang, Ming Lei, Lingbo Xu, Zeguo Tang, Can Cui, Peng Wang, Deren Yang

**Affiliations:** ^1^ State Key Laboratory of Silicon Materials and School of Materials Science and Engineering Zhejiang University Hangzhou 310027 China; ^2^ Department of Chemistry Zhejiang University Hangzhou 310027 China; ^3^ Center for Optoelectronics Materials and Devices Department of Physics Zhejiang Sci‐Tech University Hangzhou 310018 China; ^4^ Ritsumeikan Global Innovation Research Organization Ritsumeikan University, Nojihigashi Kusatsu Shiga 525‐8577 Japan

**Keywords:** fullerene interfacial layer, low‐temperature processes, perovskite solar cells, self‐organization, UV stability

## Abstract

In this Communication, a self‐organization method of [6,6]‐phenyl‐C61‐butyric acid 2‐((2‐(dimethylamino)‐ethyl) (methyl)amino)ethyl ester (PCBDAN) interlayer in between 6,6‐phenyl C61‐butyric acid methyl ester (PCBM) and indium tin oxide (ITO) has been proposed to improve the performance of N–I–P perovskite solar cells (PSCs). The introduction of self‐organized PCBDAN interlayer can effectively reduce the work function of ITO and therefore eliminate the interface barrier between electron transport layer and electrode. It is beneficial for enhancing the charge extraction and decreasing the recombination loss at the interface. By employing this strategy, a highest power conversion efficiency of 18.1% has been obtained with almost free hysteresis. Furthermore, the N–I–P PSCs have excellent stability under UV‐light soaking, which can maintain 85% of its original highest value after 240 h accelerated UV aging. This self‐organization method for the formation of interlayer can not only simplify the fabrication process of low‐cost PSCs, but also be compatible with the roll‐to‐roll device processing on flexible substrates.

Since the first perovskite solar cell (PSC) was invented by Kojima et al. in 2009, the organic–inorganic perovskite of the ABX_3_ [A = CH_3_NH_3_^+^ (MA), or NH = CHNH_3_^+^ (FA); B = Pb or Sn; X = I, Br, Cl] formula have been intensively studied because of their excellent photovoltaic properties and low‐material costs.^1^, ^2^, ^3^, ^4^ Currently, the solid‐state perovskite solar cells are quite attractive, which are usually designed as conventional (N–I–P) or inverted (P–I–N) device structures based on mesoscopic or planar heterojunctions.^5^, ^6^ Due to the efforts in morphology control,^7^, ^8^, ^9^ device architecture optimization,^10^ and interface engineering,^11^, ^12^, ^13^ a state‐of‐the‐art power conversion efficiency (PCE) of 22.1% has been achieved for the N–I–P architecture of PSCs in the lab.^14^ In this N–I–P architecture, the electron transport layer (ETL) generally adopts a mesoscopic TiO_2_ layer formed at high temperatures (>450 °C).^15^, ^16^, ^17^ Such a high temperature process is usually not favorable in the fabrication of PSCs, especially on the flexible substrates such as polyethylene terephthalate (PET). Therefore, the planar TiO_2_ without mesoscopic structure using a low‐temperature process has been attempted to be used as an ETL. Unfortunately, such a PSC based on the planar TiO_2_ has a lower efficiency than that based on the mesoscopic TiO_2_, and meanwhile suffer serious hysteresis effects.^11^, ^18^, ^19^, ^20^ Moreover, it must also be mentioned that TiO_2_ is a strong ultraviolet (UV) photocatalyst for oxidizing organic materials due to the deep‐level trap states associated with native point defects,^21^, ^22^ and therefore can degrade the perovskite on its surface under UV illumination, harmful for the stability of the PSCs.^23^, ^24^, ^25^ The introduction of interlayer such as cesium bromide can not only effectively enhance the stability of PSCs under UV‐light soaking,^26^ but also increase the fabrication complex of solar cells and meanwhile cannot eliminate the hysteresis effect of PSCs originating from the low carrier mobility in TiO_2_.

Recently, several efforts have focused on employing novel solution processed organic semiconductors as ETLs to realize low‐temperature processed PSCs.^27^, ^28^ As one of the most successful organic ETLs, fullerene (C_60_) and its derivatives also have been successfully adopted as ETLs to replace the TiO_2_ in the N–I–P architectural PSCs, which have generally been used in the P–I–N architectural devices before.^6^, ^10^, ^12^ The fullerene‐based ETLs can not only be processable at low temperatures, but also effectively mitigate the hysteresis effect of the PSCs through passivating charge trap states and defects on the surface of perovskite.^29^, ^30^ The C_60_ itself has high electron mobility and conductivity, but it is difficult to attain a uniform and full coverage C_60_ layer through a solution process, due to the low solubility of C_60_ in common organic solvents such as chloroform, chlorobenzene, etc.^31^, ^32^ Therefore, the evaporation technique based on a vacuum process with high‐energy consumption has to be used for the formation of C_60_ ETL in the fabrication of PSCs.^33^, ^34^, ^35^ Recently, metal‐oxide free N–I–P PSCs using a solution processed 6,6‐phenyl C61‐butyric acid methyl ester (PCBM) ETL have been attained and achieved a highest PCE of 15.7% with obvious hysteresis.^36^, ^37^ This value is much lower than the PCE of PSCs based on a TiO_2_ ETL, which is mainly associated with the interface barriers existing between the PCBM layer and the electrode, due to imperfect interfacial band alignment.^12^, ^36^, ^37^ Interfacial engineering by inserting a thin interlayer such as polyethylenimine, fulleropyrrolidinium iodide‐polyethyleneimine between the PCBM layer and the electrode has been employed here for reducing the interface barrier and enhancing the carrier transportation.^36^, ^37^ However, the sequential deposition of these organic interlayers and ETL on electrode always requires multistep processes, which increases the complexity of the process.^36^, ^37^ Moreover, the thickness of these semiconducting interlayers must be controlled as thin as possible (<10 nm) and meanwhile the interlayers should have full coverage in order to obtain a small series resistance and large shunt resistance for the PSCs, which is a big challenge for the traditional roll‐to‐roll techniques.

Here, we will report a self‐organization method to obtain the ETL and interlayer simultaneously by one‐step solution process, which can not only largely simplify the fabrication process, but also allow the formation of ultrathin interlayer with ease. The self‐organized interlayer can effectively decrease the interface barrier between the electrode and ETL. The resultant N–I–P PSCs can reach an efficiency of 18.1%, with almost free hysteresis. Moreover, it is verified that our PSCs exhibit excellent stability under UV light. These results pave a new way for the low‐temperature processed fabrication of N–I–P architectural PSCs with high performance and good UV stability.

The amine‐based fullerene [6,6]‐phenyl‐C61‐butyric acid 2‐((2‐(dimethylamino)‐ethyl) (methyl)amino)ethyl ester (PCBDAN) is used as an additive in PCBM to blend well in chlorobenzol solution. The mixture solution is coated on the indium tin oxide (ITO) by spin‐coating technique, as shown in **Figure** **1**a. The similar Lewis base interaction and close surface energies for ITO and PCBDAN could drive PCBDAN toward the buried ITO interface.^38^ Thus, the vertical phase separation between PCBM and PCBDAN can be realized and form an ITO/PCBDAN/PCBM structure during the spin‐coating. The surface morphology of the obtained film is quite smooth, which has uniform and full coverage on the ITO (Figure S1, Supporting Information). Figure 1b shows the cross‐sectional scanning electron microscopy (SEM) image and energy dispersive spectroscopy (EDS) mappings of PCBM:PCBDAN layer on the ITO. It can be obviously seen that there exists a uniform fullerene layer on the surface of ITO. The elemental distribution of C, N, and In can represent the positions of PCBM, PCBDAN, and ITO, respectively. The distribution of N between the C and In in the EDS mapping strongly verifies that the PCBDAN interlayer is formed between the PCBM layer and ITO.

**Figure 1:**
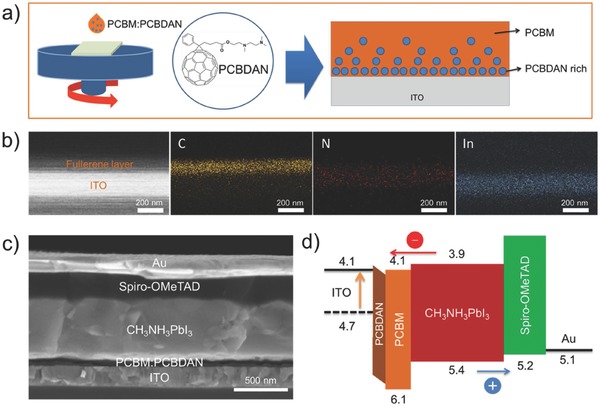
a) Formation process of the device ITO/PCBM:PCBDAN through spin‐coating. b) SEM image and EDS mapping of the cross section of ITO/PCBM:PCBDAN (≈120 nm). c) Cross‐sectional SEM image of the device with a structure of ITO/PCBM:PCBDAN/CH_3_NH_3_PbI_3_/Spiro‐OMeTAD/Au, with PCBM:PCBDAN, CH_3_NH_3_PbI_3_, and Spiro‐OMeTAD thicknesses of ≈40, 500, and 200 nm. d) Energy level diagram of the device with a self‐organized PCBDAN interlayer.

The N–I–P PSCs with a structure of ITO/PCBM:PCBDAN/CH_3_NH_3_PbI_3_/Spiro‐OMeTAD/Au have been fabricated, based on the self‐organization of PCBDAN interlayer. The cross‐sectional SEM image of an optimized PSC is shown in Figure 1c. It can be clearly observed that there is a thin and uniform fullerene layer between the ITO and perovskite layer, which is crucial for attaining hysteresis‐free and high performance device. The PCBDAN interlayer is expected to reduce the contact barrier between ITO and PCBM ETL. Figure 1d illustrates the energy band diagram of the PSCs, which highlights the variation of the work function (WF) of ITO. The ultraviolet photoelectron spectroscopy measurements on the ITO before and after PCBDAN modification, as shown in Figure S2 (Supporting Information), indicates that the WF of ITO decreases from 4.7 to 4.1 eV after the PCBDAN modification, which is well compatible with the lowest unoccupied molecular orbital of PCBM (≈4.1 eV), eliminating the interface barrier between ITO and PCBM ETL. The reduction of the WF of ITO is ascribed to the presence of a negative interfacial dipole caused by the amine functionality of PCBDAN, which can increase the electrostatic potential across the device.^39^ The strengthened electric field can enhance the extraction efficiency of carriers at the ETL/ITO interface, which effectively improves the short‐circuit current density (*J*_SC_) and fill factor (FF) of device. Moreover, the compatibility of energy bands between the ETL and ITO can increase the built‐in potential of the device, which is beneficial for maximizing the open‐circuit voltage (*V*_OC_) of PSCs.^40^, ^41^

We have first fabricated the devices with various ETL thicknesses by controlling the concentration of the PCBM chlorobenzene solution (Figure S3, Supporting Information). The typical *J*–*V* curves of the PSCs devices show that the optimal thickness for PCBM is about 40 nm (Figure S3, Supporting Information). When the PCBM layer is too thin (<25 nm), the device shows bad performance and obvious hysteresis behavior. This is probably a result of the direct contact between the perovskite and ITO (Figure S4, Supporting Information), because PCBM can be partly dissolved in precursor solution even within a short exposure time (less than 1 min) during the spin‐coating. Then, we compared the photovoltaic metrics of the PSCs with different ratio of PCBDAN/PCBM, keeping the thickness of ETLs at 40 nm **(** **Figure** **2**a–d). It can be seen that the performances of the PSCs show an increase with improving the ratio of PCBDAN only if the ratio value is smaller than 10%. When the ratio of PCBDAN/PCBM is further increased (>10%), the device performance shows a decrease. It clearly suggests that a thicker interlayer is not beneficial for the carrier transportation. In the case of 100% PCBDAN, since the highest occupied molecular orbital level (5.8 eV) of PCBDAN is shallower than that of PCBM (6.1 eV),^42^ the holes cannot be blocked more effectively and photogenerated charge recombination occurs more easily at the ETL/perovskite interface, as shown in Figure S5 (Supporting Information), which severely degrades the performance of PSCs. To make a comparison, we have also fabricated the device based on PCBDAN/PCBM bilayer through two‐step spin‐coating, which shows much worse performance than the device using PCBDAN as the additive. This is associated with the re‐dissolution of PCBDAN layer by the PCBM chlorobenzene solution during the second‐step spin‐coating process (Figure S6, Supporting Information).

**Figure 2:**
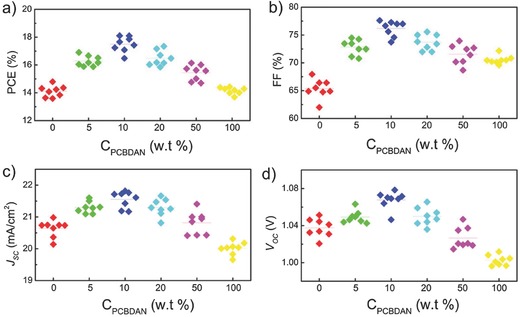
Photovoltaic metrics statistics of devices using PCBM, PCBM:PCBDAN (χ:1, χ = 19, 9, 4, 1 by weight) and PCBDAN as ETLs, respectively. a) PCE, b) FF, c) *J*_SC_, and d) *V*_OC_.

The highest current density–voltage (*J*–*V*) curve of the PSC based on a PCBM:PCBDAN ETL is shown in **Figure** **3**a. Note that the PSCs based on PCBM and TiO_2_ ETLs are used as a reference here. The detailed photovoltaic parameters are shown in **Table** **1**. It can be seen that the PSC based on the PCBM:PCBDAN ETL exhibits an efficiency of 18.1%, which is much higher than those of referenced PSCs, with a value in the range of 13%–15%. The PSC with a planar TiO_2_ ETL shows large hysteresis, consistent with most previous results, but the devices with fullerene‐based ETL show a very small hysteresis (Figure 3a). It is known that the PCBM is able to suppress the hysteresis of PSCs since the mobile ions in the perovskite could interact with PCBM to form a PCBM halide radical and then reduce electric field‐induced anion migration.^30^ Furthermore, a typical *J*–*V* curve of PSC with the PCBM:PCBDAN ETL is double‐checked with various dwelling time from 5 to 500 ms at 20 mV per step using both forward and reverse scan modes, as shown in Figure S7 (Supporting Information). The results show that the *J*–*V* curves based on different scan rates are almost same, further confirming that the device indeed has an almost free hysteresis effect. Figure 3b shows the external quantum efficiency (EQE) spectra of the PSCs with various ETLs. The integrated photocurrent densities from the EQEs are 20.23, 21.30, and 18.3 mA cm^−2^, respectively, which are consistent with the corresponding *J*–*V* measurements in Figure 3a. The steady‐state PCEs of the champion devices were measured as a function of time when the cells were biased at their respective *V*_mp_ (voltage at the maximum power point), as shown in Figure 3c. The steady‐state PCEs of the devices with the PCBM:PCBDAN, PCBM, TiO_2_ ETLs are 17.8%, 14.3%, and 11.7%, respectively. It can be seen that the steady‐state PCEs of the devices with the fullerene‐based ETLs reach the maximum value instantly, while the device with the TiO_2_ ETL needs ≈50 s mostly due to its large hysteresis. More importantly, the self‐organization method can also be applied for the fabrication of flexible solar cells. The illuminated *J*–*V* curves of PSCs with the PCBN:PCBDAN ETL on the PET substrate is shown in Figure 3d. An efficiency of 14.2% with negligible hysteresis can be attained.

**Figure 3:**
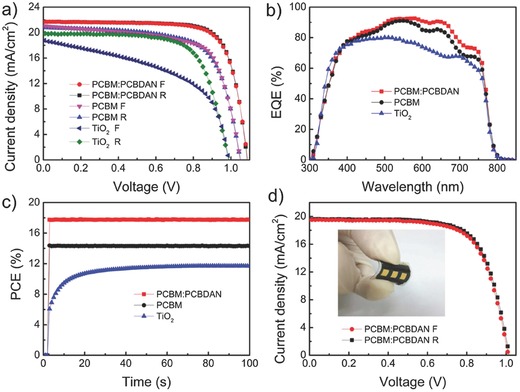
a) *J*–*V* curves b) EQE spectra of the champion PSCs with PCBM:PCBDAN, PCBM, and TiO_2_ ETL. c) Photocurrent densities measured as a function of time for the PCBM:PCBDAN, PCBM, and TiO_2_‐based devices biased at their respective *V*_mp_ 0.90, 0.82, and 0.78 V, respectively. d) *J*–*V* curve of a flexible PSC measured at different scan directions and 20 mV step widths, with dwelling time of 200 ms.

**Table 1:** Photovoltaic parameters of the PSCs with different ETLs under different scanning direction

ETLs	Scan directions	*J*_SC_ [mA cm^−2^]	*V*_OC_ [V]	FF	PCE [%]
PCBM:PCBDAN (9:1)	Reverse	21.70	1.08	77.3	18.1
	Forward	21.73	1.08	76.5	17.9
PCBM	Reverse	20.56	1.05	68.0	14.8
	Forward	20.73	1.05	67.4	14.6
TiO_2_	Reverse	19.92	0.98	68.5	13.4
	Forward	18.90	0.98	50.1	8.7
Flexible (10 wt%)	Reverse	19.77	1.02	70.4	14.2
	Forward	19.69	1.02	69.6	13.9

When contacting with charge transport layers, perovskite films can exhibit strong photoluminescence (PL) quenching, demonstrating the efficient charge transfer from the perovskite layer to the transport layer.^43^ **Figure** **4**a shows the steady‐state PL spectra of perovskite layer on TiO_2_, PCBM, and PCBM:PCBDAN. Consistent with the previous reports, the PL intensity of perovskite on the TiO_2_ layer has a lower PL quenching than that of perovskite on the PCBM.^43^ This means that electrons cannot be efficiently transferred into the TiO_2_ layer due to the lower electron mobility of TiO_2_, or the interfacial traps at the perovskite/TiO_2_ interfacial.^44^, ^45^ The PL intensity of perovskite on the PCBM:PCBDAN layer was much lower than that of perovskite on the PCBM layer, indicating that the introduction of PCBDAN can facilitate the electron extraction from perovskite layers to PCBM. This result can be supported by the well‐matched WF of ITO and the enhancement of the electron mobility after introducing the PCBDAN (Figure S8, Supporting Information). Figure 4b compares the charge carrier lifetime (τ) measured by time‐resolved photoluminescence (TRPL) in the perovskite layer on TiO_2_, PCBM, and PCBM:PCBDAN. The TRPL decay curves were fitted by assuming an exponential decay model. The values of τ are 2.0, 1.4, and 1.0 ns for the fluorine‐doped tin oxide (FTO)/TiO_2_/perovskite, ITO/PCBM/perovskite, and ITO/PCBM:PCBDAN/perovskite, respectively. It is clear that the self‐organization of PCBDAN interlayer greatly enhances the electron extraction at perovskite/PCBM interface. This can effectively improve the *J*_SC_ and FF values of PSCs.

**Figure 4:**
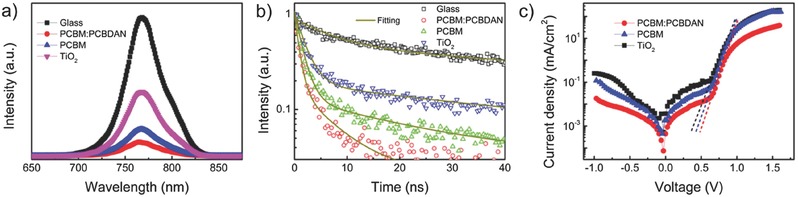
a) Steady state PL spectra and b) PL decay of perovskite thin films deposited on glass, PCBM:PCBDAN, PCBM, and TiO_2_. c) Dark *J*–*V* characteristics of devices with various ETLs.

Figure 4c shows the dark *J*–*V* characteristics of various PSCs which can be modeled by the Shockley diode equation^46^, ^47^

(1) $$J\;=\;J_{0}\;\left[\exp\;\left(\frac{qV}{\eta kT}\right)\;\;-\;1\right]$$

where *J*_0_ denotes the reverse saturation current density, *J* is the dark current density, *V* is the applied bias, η is the ideality factor, *k* is the Boltzmann constant, and *T* is the temperature. The η can be independently determined from the slope of the exponential regime of dark *J*–*V* characteristics on a semilogarithmic plot. In principle, the η equals unity in the ideal diode, whereas large charge recombination would increase the value of η for devices.^12^, ^48^ The values of η and *J*_0_ attained from these three type devices are summarized in Table S1 (Supporting Information). The results show that the η and *J*_0_ obtained from the device with a PCBM ETL are 1.58 × 10^−7^ and 3.24 × 10^−7^ mA cm^−2^, respectively, which are both lower than those of the device with a TiO_2_ ETL (1.72 × 10^−6^ and 4.21 × 10^−6^ mA cm^−2^). After self‐organizing the PCBDAN interlayer onto the ITO, the values of η and *J*_0_ of device further decrease to 1.30 × 10^−8^ and 1.48 × 10^−8^ mA cm^−2^, respectively. It suggests that the self‐organized PCBDAN interlayer between ITO and ETL can effectively reduce the charge recombination loss, which is consistent with the previous reports.^12^, ^41^ Therefore, the *V*_oc_ and FF values of PSCs are significantly improved, due to the self‐organization of PCBDN interlayer.

In order to compare the UV stability of the devices with the fullerene‐based (PCBM:PCBDAN = 9:1) and TiO_2_ ETL, we exposed the two type of unpackaged cells to constant 365 nm 190 mW cm^−2^ UV illuminations in an N_2_ atmosphere, which were taken out and measured every 12 h in air with ≈45% humidity. It is worth noting that the intensity of UV light with a power of 190 mW cm^−2^ is almost equivalent to that of 41 suns. **Figure** **5**a shows the normalized PCEs as a function of testing time for the devices with PCBM:PCBDAN and TiO_2_ ETLs, respectively. Under the UV‐light stress, the PCE of device with a TiO_2_ ETL shows a constant drop during the UV‐induced aging test and lose ≈70% of its initial value within 240 h. Encouragingly, the device with a PCBM:PCBDAN ETL was found to be extremely stable: the PCE shows a slight degrade only within 12 h and then maintains about 85% of its original highest value after another 228 h (Figure 5a). The normalized *V*_OC_, *J*_SC_, and FF of the two type devices are present in Figure 5b–d, which show the same trend as the PCE values.

**Figure 5:**
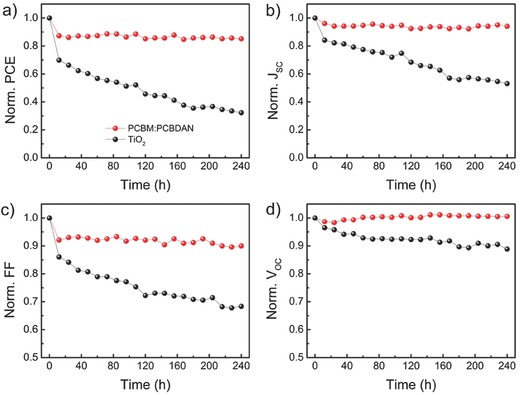
Device performances of the devices with PCBM:PCBDAN and TiO_2_ ETL exposed in N_2_ glovebox under constant 190 mW cm^−2^ UV irradiation and measured in air (≈45% humidity and 25 °C) every 12 h. Normalized a) PCE, b) *J*_SC_, c) FF, and d) *V*_OC_.

To better understand the mechanisms of degradation, the X‐ray diffraction (XRD) of the devices with the PCBM:PCBDAN and TiO_2_ ETLs were checked during the UV aging. As shown in Figure S9 (Supporting Information), a new diffraction peak around 12.6° corresponding to PbI_2_ are observed in the XRD pattern for the device with a TiO_2_ ETL after UV aging, which is related to the decomposition of perovskite induced by the TiO_2_. The PbI_2_ at the perovskite/TiO_2_ interface would block the electron transport, resulting in the decrease of *J*_SC_ (Figure 5b) and the increase of *R*_S_ (Figure S10, Supporting Information). Thus, the FF of the device with a TiO_2_ ETL shows a decrease after the UV irradiation (Figure 5c). In comparison, there are no PbI_2_ diffraction peaks emerging for the device with a PCBM:PCBDAN ETL during the whole UV‐aging process, which shows good stability (Figure S9, Supporting Information). The X‐ray photoelectron spectroscopy (XPS) and UV–vis absorption spectroscopy of PCBM:PCBDAN layer before and after UV illuminations were measured (Figures S11 and S12, Supporting Information). The negligible changes of XPS and UV–vis spectrum indicate that the PCBM:PCBDAN layer exhibits high stability under UV aging in inert atmosphere. In addition, we exposed the FTO/TiO_2_/MAPbI_3_ and ITO/PCBM:PCBDAN/MAPbI_3_ in air under 418 mW cm^−2^ UV irradiation for 4 h (Figure S13, Supporting Information). An obvious difference in color degradation owing to perovskite decomposition became clearly visible, indicating that the TiO_2_ can indeed induce the decomposition of perovskite under UV‐light exposure. These stress tests suggest that the devices with PCBM:PCBDAN ETLs are stable over long‐term operation against UV‐light soaking, in contrast to the devices with TiO_2_ ETLs.

In summary, we have proposed a self‐organization method of PCBDAN interlayer inbetween PCBM and ITO to improve the performance of N–I–P PSCs. The introduction of self‐organized PCBDAN interlayer can effectively reduce the work function of ITO and therefore eliminate the interface barrier between ETL and electrode. It is beneficial for enhancing the charge extraction and decreasing the recombination loss at the interface. By employing this strategy, we have obtained a highest PCE of 18.1% with almost free hysteresis. Furthermore, our N–I–P PSCs have excellent stability under UV‐light soaking, which can maintain 85% of its original highest value after 240 h accelerated UV aging. This self‐organization method for the formation of interlayer can not only simplify the fabrication process of low‐cost PSCs, but also be compatible with the roll‐to‐roll device processing on flexible substrates.

## Experimental Section

*Perovskite Solar Cell Fabrication*: The ITO was cleaned sequentially in deionized water, cleaning fluid, acetone, and ethanol under sonication for 5 min, respectively. Then the ITO was dried by nitrogen gas and treated with UV‐Ozone machine for 20 min. PCBDAN was synthesized with literature method.^49^ PCBM, PCBM:PCBDAN (χ:1, χ = 19, 9, 4, 1 by weight) blend, and PCBDAN materials were dissolved in chlorobenzol solution, respectively. The ETL layer on ITO with thicknesses of ≈25, 40, 60, 80 nm was attained by spin‐coating the chlorobenzol solution with concentrations of 10, 12.5, 15, and 17.5 mg mL^−1^ at a rate of 2000 rpm for 60s, respectively. The 120 nm ITO/PCBM:PCBDAN sample for EDS measurements was attained by spin‐coating the chlorobenzol solution of PCBM:PCBDAN (9:1 by weight) with 25 mg mL^−1^. For the PCBDAN/PCBM bilayer, 2 mg mL^−1^ PCBDAN and 12.5 mg mL^−1^ PCBM chlorobenzol solution were spin‐coated on ITO substrates at 2000 rpm successively. The ITO/ETL layer was then followed by thermal annealing at 100 °C for 10 min. For planar TiO_2_ ETL, an acidic solution of titanium isopropoxide in ethanol (the concentrations of titanium isopropoxide/2 m HCl/ethanol = 254 µL/34 µL/2 mL) was spin‐coated on the FTO at 2000 rpm for 40 s, and then annealed in air at 500 °C for 30 min. The 461 mg of PbI_2_, 159 mg of CH_3_NH_3_I, and 78 mg of DMSO (molar ratio 1:1:1) were mixed in 600 mg of dimethyl formamide (DMF) solution. The prepared solution was dropped on different substrates and then rapidly spin‐coated at 1000 rpm for 10 s and 5000 rpm for another 20 s. 0.6 mL of diethyl ether was drop‐casted quickly 15 s before the 5000 rpm spin‐coating ended. The perovskite films were heated at 70 °C for 1 min and 100 °C for 10 min on a hotplate, respectively. After several minutes, a hole‐transport material was spin‐coated on the top of perovskite film at the rotation speed of 3000 rpm for 30 s in glovebox. The hole‐transport solution was prepared by dissolving 72.3 mg spiro‐MeOTAD, 17.5 mL of a stock solution of 520 mg mL^−1^ lithium bis(trifluoromethylsulphonyl)imide in acetonitrile and 28.8 mL 4‐tert‐butylpyridine in 1 mL chlorobenzene. At last, 100 nm thick Au electrode were deposited under high vacuum (<1.0 × 10^–3^ Pa). For all devices, the active area is 10 mm^2^. For UV‐stability measurement, the perovskite solar cells with PCBM:PCBDAN and TiO_2_ ETLs were aged under 365 nm 190 mW cm^−2^ UV illuminations in N_2_ atmosphere. All the devices were unpaged and measured in air with ≈45% humidity.

*Materials and Equipment*: Lead iodide (PbI_2_, 99%), PC_61_BM (99.5%), dimethylsulfoxide (DMSO, 99.9%), chlorobenzol (CB, 99.8%), and DMF (99%) were purchased from Sigma‐Aldrich. Spiro‐OMeTAD and CH_3_NH_3_I was purchased from Xi'an Polymer Light Technology Corporation. Diethyl ether was purchased from Sinopharm Chemical Reagent Co., Ltd. The current density versus voltage (*J*–*V*) characteristics of the solar cell were measured by a Keithley 2400 source meter with a solar simulator (94022A, Newport) under AM 1.5 G conditions at an illumination intensity of 100 mW cm^−2^, calibrated by a standard Si solar cell (PVM937, Newport). The SEM data were scanned by an S‐4800 (Hitachi) field‐emission scanning electron microscope. The TRPL spectroscopy was measured with the PL spectrometer (Edinburgh Instruments, FLS 920). The samples were excited by a pulsed laser, with a wavelength and frequency of 635 nm and 1 MHz. A band pass filter at 655 nm was used to filter out the excitation light in the transient PL measurements. The EQE of solar cells was measured by an EQE measurement system (Model QEX10, PV Measurements, Inc.) across a wavelength range of 300–850 nm. UV stability was measured using an M‐Ultra violet Light Source (MUA‐165).

## Supporting information

Supplementary
