# Supplementary material for: Self‐Organized Fullerene Interfacial Layer for Efficient and Low‐Temperature Processed Planar Perovskite Solar Cells with High UV‐Light Stability
Source: Adv Sci (Weinh). 2017 Apr 19;4(8):1700018. doi: 10.1002/advs.201700018 (PMC5566248; doi:10.1002/advs.201700018)
Supplement: Supplementary file 1 — Supplementary [file ADVS-4-na-s001.pdf]

Copyright WILEY-VCH Verlag GmbH & Co. KGaA, 69469 Weinheim, Germany, 2017.

ADVANCED SCIENCE
Open Access

Supporting Information

for *Adv. Sci.,* DOI: 10.1002/advs.201700018

Self-Organized Fullerene Interfacial Layer for Efficient and Low-Temperature Processed Planar Perovskite Solar Cells with High UV-Light Stability

Jiangsheng Xie, Xuegong Yu, Jiabin Huang, Xuan Sun, Yunhai Zhang, Zhengrui Yang, Ming Lei,* Lingbo Xu, Zeguo Tang, Can Cui, Peng Wang, and Deren Yang**

Supporting Information

Self-Organized Fullerene Interfacial Layer for Efficient and Low-temperature Processed Planar Perovskite Solar Cells with High UV-light Stability

Jiangsheng Xie[a], *Xuegong Yu*[a*], *Jiabin Huang*[a], *Xuan Sun*[b], *Yunhai Zhang*[c], *Zhengrui Yang*[a], *Ming Lei*[b*], *Lingbo Xu*[c], *Zeguo Tang*[d], *Can Cui*[c], *Peng Wang*[b], *Deren Yang* [a*]

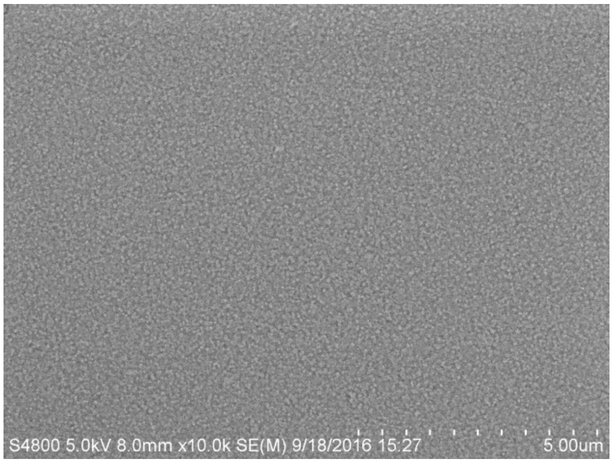

Figure S1. SEM of the ITO/PCBM:PCBDAN layer surface.

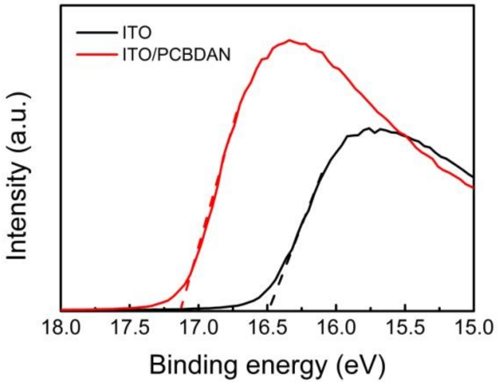

Figure S2. UPS spectra of the ITO and ITO/PCBDAN

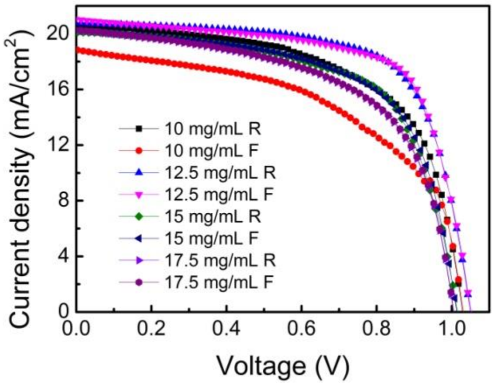

Figure S3. Typical *J-V* curves of the PSCs at different scan directions with different thickness of PCBM layer. 25, 40, 60, and 80 nm were obtained by using PCBM concentrations of 10, 12.5, 15, and 17.5 mg mL^{-1}, respectively

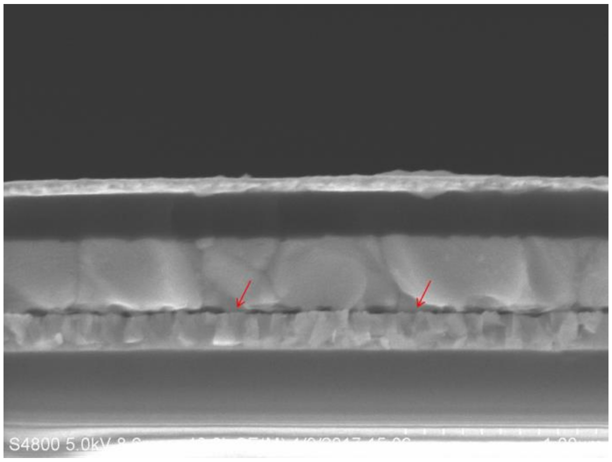

Figure S4. Cross-sectional SEM image of the device in which the PCBM layer was attained by spin-coating the chlorobenzol solution with concentrations of 10 mg mL^{-1}.

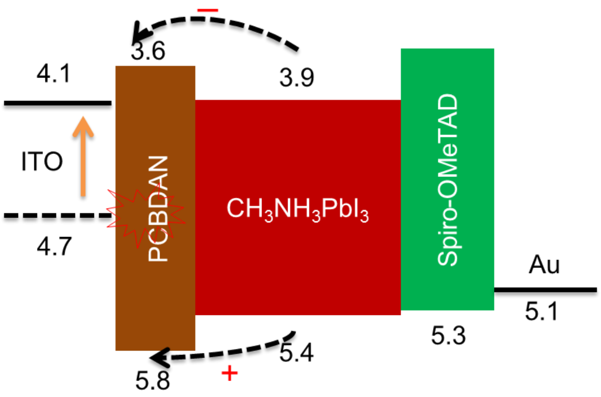

Figure S5. Energy level diagram of the device with structure of ITO/PCBDAN/$CH_3NH_3PbI_3$/Spiro-OMeTAD/Au

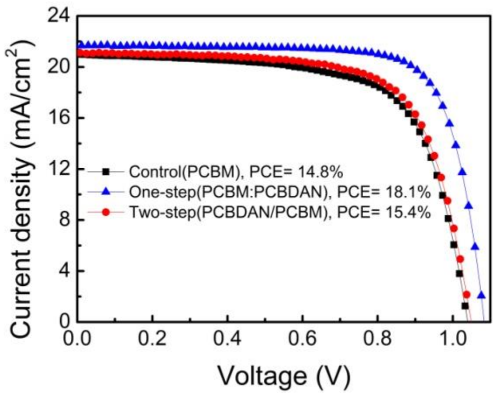

Figure S6. Typical *J-V* curves of the PSCs based on different ETLs.

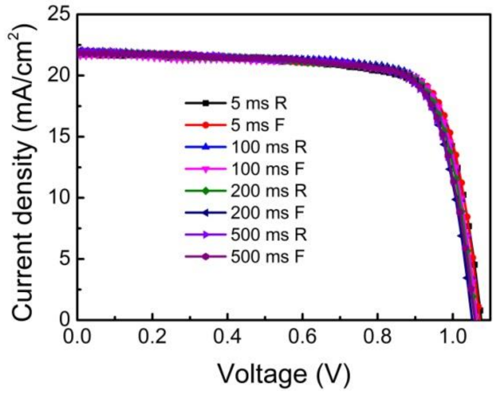

Figure S7. *J–V* curves of a typical PSC measured at the forward scan and the reverse scan with various dwelling times (5 ms to 500 ms) at 20 mV per step.

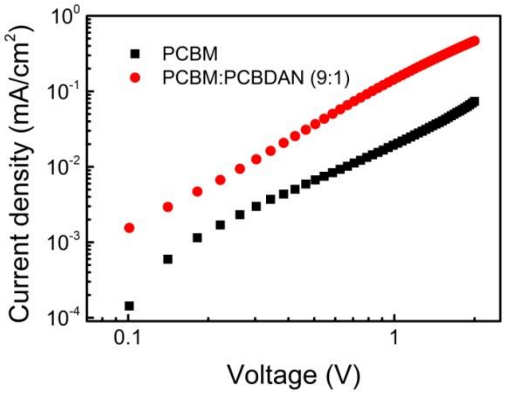

Figure S8. *I–V* measurement of the electron-only devices with the structure of ITO/ZnO/PCBM or PCBM:PCBDAN (9:1)/Ag.

Table S1. η and J_0 from dark J–V measurements of the device with different ETL.

ETL	η	J_0 (mA cm^{-2})
TiO_2	1.72	4.21×10^{-6}
PCBM	1.58	3.24×10^{-7}
PCBM:PCBDAN (9:1)	1.30	1.48×10^{-8}

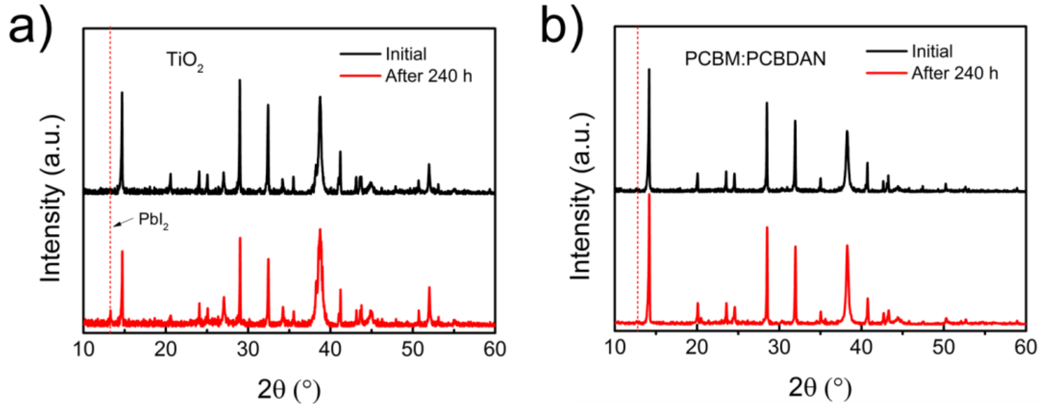

Figure S9. XRD patterns of PSCs a) with TiO_2 and d) PCBM:PCBDAN ETL before and after 190 mW cm^{-2} UV aging.

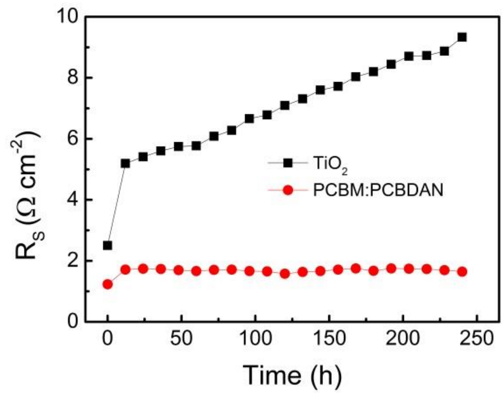

Figure S10. R_S of the device with different ETLs exposed in N_2 glovebox under constant 190 mW cm^{-2} UV irradiation.

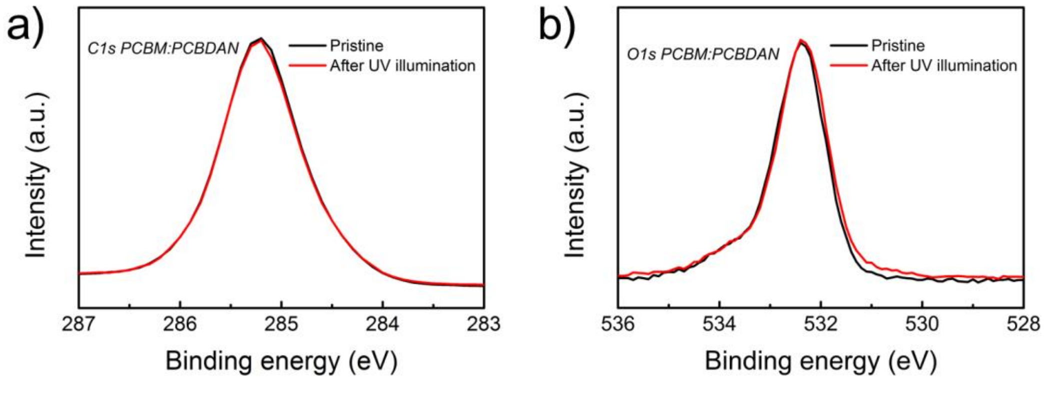

Figure S11. XPS spectra of ITO/PCBM:PCBDAN sample before and after UV illumination.

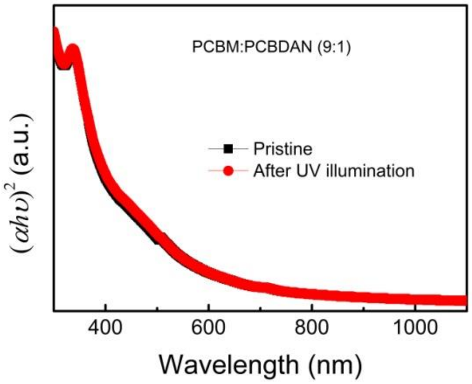

Figure S12. UV−Vis spectra of ITO/PCBM:PCBDAN sample before and after UV illumination.
